# A New Secondary Structure Assignment Algorithm Using C_α_ Backbone Fragments

**DOI:** 10.3390/ijms17030333

**Published:** 2016-03-11

**Authors:** Chen Cao, Guishen Wang, An Liu, Shutan Xu, Lincong Wang, Shuxue Zou

**Affiliations:** 1 College of Computer Science and Technology, Jilin University, Changchun 130012, China; caochen_jlu@hotmail.com (C.C.); gcwang13@mails.jlu.edu.cn (G.W.); xushutan@hotmail.com (S.X.); wlincong@hotmail.com (L.W.); 2 Key Laboratory of Symbol Computation and Knowledge Engineering of the Ministry of Education, Jilin University, Changchun 130012, China; 3 College of Pharmaceutical Science, Jilin University, Changchun 130012, China; anran1222@163.com

**Keywords:** protein, secondary structure assignment, cluster, C_α_ backbone fragment, outlier detection

## Abstract

The assignment of secondary structure elements in proteins is a key step in the analysis of their structures and functions. We have developed an algorithm, SACF (secondary structure assignment based on C_α_ fragments), for secondary structure element (SSE) assignment based on the alignment of C_α_ backbone fragments with central poses derived by clustering known SSE fragments. The assignment algorithm consists of three steps: First, the outlier fragments on known SSEs are detected. Next, the remaining fragments are clustered to obtain the central fragments for each cluster. Finally, the central fragments are used as a template to make assignments. Following a large-scale comparison of 11 secondary structure assignment methods, SACF, KAKSI and PROSS are found to have similar agreement with DSSP, while PCASSO agrees with DSSP best. SACF and PCASSO show preference to reducing residues in N and C cap regions, whereas KAKSI, P-SEA and SEGNO tend to add residues to the terminals when DSSP assignment is taken as standard. Moreover, our algorithm is able to assign subtle helices (3_10_-helix, π-helix and left-handed helix) and make uniform assignments, as well as to detect rare SSEs in β-sheets or long helices as outlier fragments from other programs. The structural uniformity should be useful for protein structure classification and prediction, while outlier fragments underlie the structure–function relationship.

## 1. Introduction

In 1951, Pauling and colleagues first defined two main secondary elements (α-helix and β-sheet) based on the intra-backbone hydrogen bond patterns in proteins [1]. They correctly detected the idealized π-helix but incorrectly predicted that 3_10_-helix would not occur due to unfavorable angles. However, approximately 4% of residues in proteins have been shown to occur in this secondary element [2]. Except for the two predominant secondary structure elements and two helical elements, other minor secondary structural elements (SSE) such as β-turns [3], β-bulges [4], γ-turns [5] and loops have been defined using the hydrogen bond information in proteins. All SSEs are usually grouped into three larger classes: helix, strand and coil [6]. To date, secondary structures have been extensively employed in structure visualization [7], classification [8], comparison [9], and prediction [10].

The first SSE assignment program, proposed by Levitt and colleagues, automatically detected SSEs using C_α_ distance, inter-C_α_ torsion angle and peptide hydrogen bond patterns [11]. DSSP was subsequently developed and has become the most popular program in the field, serving as the “gold standard” [12]. Moreover, most SSE prediction methods are based on DSSP assignments [13], which identifies backbone hydrogen bond patterns based on an electrostatic approximation of hydrogen bond energy followed by SSE assignment using hydrogen bond pattern information. STRIDE, which is the second most popular algorithm at present, employs a modified hydrogen bond energy function and the statistical probability factors of main-chain dihedral angles derived from Protein Data Bank (PDB) [14] records to perform SSE assignments [15]. SECSTR is a new addition to the DSSP program that is dedicated to identifying π-helices, which were seldom assigned by older versions of DSSP and STRIDE [16].

In addition to the aforementioned programs, which assign SSEs by detecting hydrogen bond information between backbone atoms, more than a dozen geometry-based SSE assignment programs have been developed. Geometry-based secondary structure assignment programs can be generally categorized into two groups: (1) methods that use the geometrical restraint of local fragments and (2) methods that fit C_α_ coordinates to a line or curve. P-SEA uses a short-range C_α_ distance mask (*i* to *i* + 2, *i* + 3 and *i* + 4) and two dihedral angle criteria for secondary structure assignment [6]. KAKSI develops an assignment by defining allowed C_α_ distance measures and dihedral angles [17]. Similar to P-SEA, XTLSSTR also calculates three distances and two backbone dihedral angles to determine SSE, but two distances are H-bond distances instead of C_α_ distances [18]. PALSSE delineates SSEs from C_α_ coordinates and uses distance as well as torsion angle restraints to detect core elements; core elements are then extended to longer fragments [19]. SABA introduces a novel geometrical parameter, a pseudo center, which is the midpoint of two continuous C_α_s, and assigns SSEs using cut-off criteria for distances as well as dihedral angles of two or more pseudo centers and C_α_ atoms [20]. PROSS defines SSEs based solely on backbone torsion angles [21], whereas SENGO uses the angle between successive peptide bonds for helix assignment and backbone dihedrals as well as alternating peptide bonds for β-sheet assignment [22]. More recently, DISICL and PCASSO have been developed. DISICL classifies SSEs into 18 distinct classes based solely on the main-chain dihedral angles of two consecutive residues; PCASSO applies Random Forests in learning 258 geometric features calculated by C_α_s and pseudo centers (see SABA) at different positions [23,24].

Several other programs can be classified into the second category. DEFINE assigns SSEs by matching C_α_ coordinates with a linear distance matrix of ideal secondary structures [25]. STICK, which is considered a variant of DEFINE, fits a set of line segments independent of any external secondary structure definition to avoid the problem of fitting a single line to a bent structure [26]. SSE assignment in P-CURVE is based on matching a peptide backbone to motifs that have idealized helical parameters and generates a global curved axis [27]. In particular, SKSP and PSSC do not belong to any category mentioned before: SKSP performs SSE assignments by averaging four popular programs: STRIDE, KAKSI, SECSTR and PSEA [28]; PSSC uses DSSP output and introduces detailed eight-character secondary structure information to characterize protein structures [29].

In general, the majority of geometry-based methods exhibit a broad consensus at most helix and strand core segments in proteins. For KAKSI, the agreement with DSSP is 91.7% and 92.1% for helices and strands, respectively, whereas the agreement between P-SEA and DSSP for the two major elements is 93.8% and 78.4% [6,17]. The main difficulties for secondary structure assignment can be categorized into three areas: (1) locating the terminus of the helix/strand; (2) distinguishing distortions and breaks in the secondary structure [17]; and (3) detecting and prioritizing subtle secondary structures, such as 3_10_-helices and π-helices. As DSSP recognizes SSEs well and agrees with intuitive visual criteria [15], irregular and outlier fragments assigned by DSSP need to be distinguished, and the remaining “regular” fragments may serve as templates for new SSE assignments to make the assignments more uniform and visually acceptable. To address this problem, we developed a method SACF that assigns SSEs in three steps: First, outlier SSE fragments are detected. Next, the central fragments are derived by clustering the remaining fragments. Finally, new SSE fragments are assigned by aligning them to the template central fragments. An outlier SSE fragment is one that is far away from its *k*-nearest neighbor fragments. SSE fragments are often closely packed together. Thus, an outlier SSE fragment is irregular compared with its neighbors. A central SSE fragment is a fragment that has the minimum total RMSD compared to all other fragments within a cluster. Instead of only excluding local outlier torsional angles (ϕ/ψ) as STRIDE does [15], our method focuses on whole C_α_ fragments and addresses irregular SSEs. Several methods have been proposed for capturing outliers [30] and performing data clustering [31]. In the present study, a geometric clustering algorithm [32] proposed by us was applied to the clustering process, whereas a local distance-based outlier factor (*LDOF*) was used in the outlier fragment detection process [33]. The central fragment in each cluster served as a template fragment, and accurate assignment to a particular type is made based on a smaller root-mean square deviation (RMSD) than the threshold after alignment to the template fragment. We assumed that the best method should uniformly assign secondary structures, meaning that the same secondary structures should be aligned with minimum RMSD. Our method does not utilize hydrogen bonds, backbone dihedral angles, backbone NH or CO coordinates, or virtual bond lengths or angles. The program SACF is available upon request.

More than 20 SSE assignment methods have been developed; however, only Martin *et al.* undertook a comparison for six SSE assignment methods [17] and Colloc’h compared three methods: DSSP, P-CURVE and DEFINE [34]. Moreover, the agreement measures were inconsistent across different papers. We applied our algorithm to identify helices and β-sheets in the protein set and compared our assignments with 10 available programs that employ different criteria for SSE assignment: DSSP, STRIDE, P-SEA, KAKSI, DISICL, PALSSE, SEGNO, PROSS, XTLSSTR and PCASSO. The comparisons were performed based on two X-ray protein databases with middle and low resolution, as well as with NMR protein structures. We also discuss the N and C cap region of different SSE assignment methods, as most disagreements between different methods arise in the terminal regions of the assigned SSEs [13,28,34].

## 2. Results and Discussion

Set **T** consists of 2817 structures with resolutions between 2.0 and 3.0 Å, which was selected to compare our method with ten other programs, including two hydrogen bond-based SSE assignment programs (DSSP and STRIDE) and nine geometry-based methods. As shown in Table 1, twelve pairs of programs share a *Q3* score of more than 84% (bold). The agreement between the nine geometry-based methods and two hydrogen bond-based methods ranged from 72.9% to 93.5%, whereas the range of agreement among the geometry-based methods was wider, from 63.1% to 86.2%. Notably, all of the SSEs are generally grouped into three categories (helix, strand, and coil) because most geometry-based methods do not provide subtle secondary structure types. In summary, SACF agrees better with DSSP and STRIDE (84.7% and 85.1% respectively) than with other geometry-based methods except PCASSO. PCASSO achieves high agreement with DSSP (93.5%) because the protein secondary structures in the training set were assigned by DSSP and 258 geometric features were used in random decision forests. KAKIS and PROSS have similar *Q3* scores with DSSP; the agreement between these two methods and DSSP is 83.5% and 84.3%. DISICL and PALSSE assignment results are very different from the other methods. We also provide a comparison of the 11 methods on set **L** and set **N** (Tables S1 and S2); the results show that these methods share similar *Q3* scores with DSSP on set **L**, except for PCASSO, with a *Q3* score of 93.5% on set **T** and a *Q3* score of 88.1% on set **L**. Konagurthu reported that the agreement of β-strand between DSSP and STRIDE for NMR proteins was rather poor [13]; however, we found that these two methods show similar agreement with β-strands for the NMR structures.

*SOV* scores are usually employed to evaluate secondary structure predictions, but this criterion can also be applied between two structure assignments [17]. The *SOV* score value is dependent on which method is selected as the reference assignment result; we take each method as the reference in turn. As shown in Table 2 and Table 3, we computed *SOV* scores between any two of the 11 SSE assignment methods for helix and β-sheet.

For helix comparison, when the SACF assignment result is taken as the reference, the highest *SOV* score is obtained with DSSP (96.6%), followed by PCASSO (95.2%). If the DSSP assignment result is taken as the reference, PCASSO achieves an *SOV* score of 94.1% compared with DSSP, with an *SOV* score of 93.7% between STRIDE and DSSP. SACF yields an *SOV* score of 91.3% with DSSP, while KAKSI and PROSS show similar *SOV* scores with DSSP compared with SACF. When DISICL and PALSSE are selected as references, the *SOV* scores between other methods and these two methods are relatively low, ranging from 72.8% to 89.9% for DISICL and from 47.8% to 69.0% for PALSSE.

For β-sheet segment comparison, *SOV* scores are lower compared with helix, as β-sheets are more irregular than helices [34]. SACF, KAKSI, SEGNO, and PCASSO achieve *SOV* scores of 81.2%, 88.0%, 80.4% and 89.2%, respectively, compared with DSSP as the reference method. For a given reference assignment in SACF, the *SOV* scores between SACF and four methods (DSSP, STRIDE, KAKSI, PCASSO) are very close. Similar to helix, DISICL and PALSSE show very poor *SOV* scores compared with the other methods.

In conclusion, SACF, KAKSI, and PROSS show similar agreement with DSSP, while a higher agreement is seen between PCASSO and DSSP. Among the four methods SACF, KAKSI, PROSS and PCASSO, only SACF divides helix into three sub secondary elements: α-helix, 3_10_-helix, π-helix and left-handed helix. The aim of SACF is to make the secondary structure elements more uniform, and every element has its unique C_α_ fragment conformation; thus, some irregular β-sheet elements assigned by DSSP, such as β-bulge and β-hairpin, are selected as outliers by the outlier detection process of our algorithm, as these elements are short, rare and have similar C_α_ conformations with other elements such as loops and turns in proteins.

**Figure 1:**
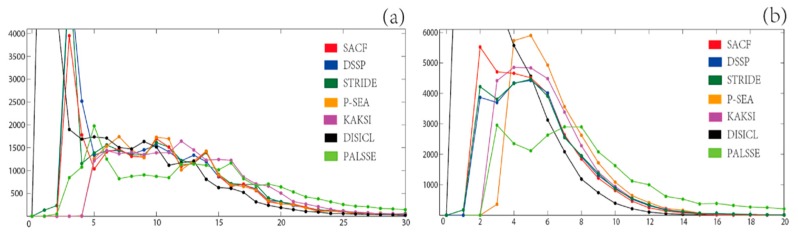
The distribution of the lengths of helices (**a**) and β-sheets (**b**) from SACF and the other six methods on set **T**. The x-axis represents helix length (**a**) or β-strand length (**b**), while the y-axis represents the number of secondary structures of that particular length.

The length distributions of helices and strands assigned by SACF, DSSP, STRIDE, P-SEA, KAKSI, DISICL, and PALSSE on set **T** are shown in Figure 1. The average number of residues are 10.19 (SACF), 9.31 (DSSP), 9.61 (STRIDE), 11.64 (P-SEA), 12.53 (KAKSI), 5.90 (DISICL), and 13.67 (PALSSE) for helix and 4.69 (SACF), 5.38 (DSSP), 5.36 (STRIDE), 6.38 (P-SEA), 5.88 (KAKSI), 3.05 (DISICL), and 9.32 (PALSSE) for strand in β-sheet. DISICL assigns a large number of 1-residue-long helices (11,605) and 1-residue-long strands in β-sheet (16,123), which are not shown in Figure 1. The distribution of the number of residues per helix has a jagged curve around 4 or 5 residues, except for DISICL and KAKSI. KAKSI provides the second highest number of long helices (more than 15 residues), while SACF, DSSP, STRIDE, and P-SEA assign very similar length distributions for helices of more than 12 residues. SACF assignment results in a slightly smaller number of 3-residue-long helices than both DSSP and STRIDE, whereas P-SEA and KAKSI do not assign helices shorter than 5 residues.

In the β-strand distribution, SACF assigns a larger number of strands with 2 to 3 residues than DSSP and STRIDE, as we provide a β-sheet ladder matching step for single strands. In the range of 4 to 7 residues, small differences are observed between SACF, DSSP and STRIDE; however, P-SEA and KAKSI show larger numbers of SSEs in this scope. For the zone of more than 8 residues in length, PALSSE assignment results in the largest number of strands in β-sheet, followed by P-SEA. In this range (length >8 residues), DSSP and STRIDE assign more strands in β-sheet than does SACF.

The capping regions show the most differences between different SSE assignment methods [17]. If we take the cap regions defined by DSSP as the standard, we search the positions corresponding to the N and C caps of DSSP with other methods. Analyses of the N and C caps defined by DSSP and other methods are shown in Table 4 and Table 5. Seven methods, including STRIDE, SACF, P-SEA, KAKSI, SEGNO, PROSS, and PCASSO, have an overall agreement of more than 80% with DSSP, but the number of helices identical to DSSP are diverse. STRIDE assignment results in 11,388 helices identical to DSSP, as they both apply a hydrogen bond pattern in SSE assignment. P-SEA and KAKSI only have 1639 and 1761 helices, respectively, that are identical to the DSSP assignment results, while these numbers for SACF and PCASSO are 5194 and 5950, respectively. P-SEA, KAKIS and SEGNO tend to extend the C cap and N cap compared with DSSP assignment. By contrast, SACF and PCASSO prefer to reduce both cap regions.

Compared with assigning the extremities of helices, the N cap and C cap of β-sheet assigned by other methods (except STRIDE) are more inconsistent with DSSP. Similar to helix, SACF and PCASSO prefer to reduce both the N and C cap regions by one or two residues compared with DSSP, whereas P-SEA, KAKSI and SEGNO are more likely to add one or two residues to both terminals of helices and β-sheets defined by DSSP. The residues located in the cap region defined by DSSP but reduced by SACF indicate that the C_α_ fragments of these residues are irregular and detected as outliers although their backbone atoms can form hydrogen bonds in the DSSP SSE assignment standard.

**Figure 2:**
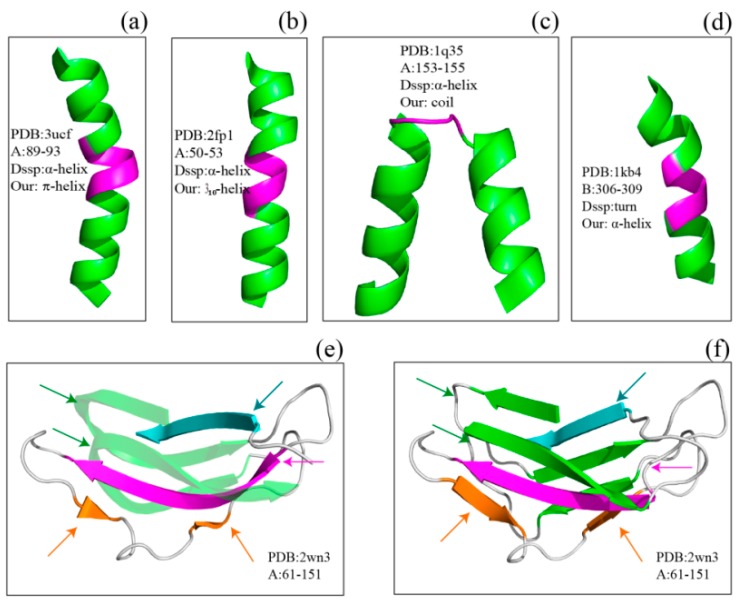
Examples of disagreement between SACF and DSSP. (**a**–**d**) show difference in helix assignment between SACF and DSSP while (**e**,**f**) illustrate the difference in β-sheet. The divergently assigned regions are shown in magenta in the top four panels and are labeled with arrows in the bottom two panels. The PDB ID and residue number are labeled in the figures, and we also provide the hydrogen bond information for (**a**,**b**) (Figure S1).

**Figure 3:**
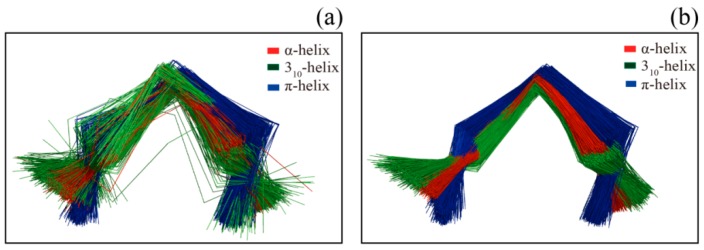
The 5-residue-long fragments assigned by DSSP (**a**) and SACF (**b**). Three helix elements (α-helix, 310-helix and π-helix) are involved in the figure. We randomly selected 1000 fragments for the three helix elements assigned by DSSP (**a**) and SACF (**b**). As can be seen, the three helix elements assigned by SACF can be better separated compared with DSSP assignment.

Figure 2 shows several examples of disagreement between our method and DSSP. The agreement between our method and DSSP for π-helices is better than that for 3_10_-helices; the π-helices we assigned were more uniform, and their geometry differed from that of α-helices (Figure 2a and Figure 3). The top four panels of Figure 2 illustrate the subtle differences in helix assignment. Although 3_10_-helices are not easily distinguished from α-helices because their C_α_-fragment poses are so similar, we continued to be able to identify fragments that should only match 3_10_-helices (Figure 2b). Specifically, the 3_10_-helix-forming (*i*, *i* + 3) hydrogen bond energy is also stronger than the α-helix-forming (*i*, *i* + 4) hydrogen bond energy at this fragment according to the DSSP output (Figure S1). The C_α_ fragments of three helices (α-helix, 310-helix and π-helix) assigned by SACF are more uniform and can be clearly separated, whereas the C_α_ fragments of the three helices assigned by DSSP show some intersection (Figure 3). Figure 2c,d describe the disagreement in α-helix assignment. Because the merging process and kink pose in our method are selected based on their incidence in the DSSP assignment, a long helix assigned by DSSP is divided into two individual helices in our assignment (Figure 2c), and two helices assigned by DSSP are “merged” into a single helix because the fragment between the two helices can be matched to our central helix poses.

The bottom two panels in Figure 2 show examples of the disagreement in β-sheet assignment between our method and DSSP. Our method often splits kinked β-strands or β-strands accompanied by β-bulges assigned by DSSP into two or more structures because the curved part of the β-strand does not match our central β-strand poses. The residues establish hydrogen bonds with their pairs but do not match the β-strand central poses.

## 3. Methods

### 3.1. The Data Set

Set **A**: Set **A** consists of 9898 X-ray proteins with a maximum R-factor of 0.2; any two structures in set A have at most 30% sequence identity. 

We divided set **A** into three subsets according to the resolution of the structure: Reference set (set **R**, resolution less than 2.0 Å, 6961 proteins), Testing set (set **T**, resolution between 2.0 and 3.0 Å, 2817 proteins), and Low-resolution protein set (set **L**, resolution more than 3.0 Å, 120 structures).

Set **N**: Set **N** contains 2233 NMR proteins with less than 30% sequence identity; each structure has at least one helix and one β-sheet according to the PDB website advanced search [35]. For NMR entities containing several models, only the first model in the PDB file was used for comparison.

### 3.2. Secondary Structure Assignment by DSSP

Secondary structural features in set **R** were assigned by DSSP, which is arguably the most popular secondary structure assignment program at present. Because the currently available version of DSSP (version 2.2.1) does not label the handedness of 3_10_-helices and α-helices, left-handed helix assignment criteria (the ϕ of the residues in the left-handed helix fell between 30° and 130°, and the ψ of the residues lie between −50° and 100°) proposed by Novotny and Kleywegt [36] was employed for left-handed 3_10_-helix and left-handed α-helix detection. Notably, the length for helix in this paper was extended by two terminal residues, *i.e.*, for a helix fragment (residue *i* to *j*) assigned by DSSP, the residues *i* − 1 and *j* + 1 were both considered to be involved in the helix, as the two residues also establish hydrogen bonds with residues in the helix according to the hydrogen bond pattern definition of DSSP. Hence, the minimal lengths for 3_10_-helices, α-helices and π-helices are 5, 6, and 7 residues, respectively.

### 3.3. Outlier Detection

*LDOF* [33] was used to detect outlier fragments. This algorithm uses the relative location of a fragment with respect to its neighbors to determine the degree to which the fragment deviates from its neighborhood. Fragments with high *LDOF* values indicate that the pose deviates more from its nearest neighbors and are more likely to be an outlier fragment. The local distance-based outlier factor *x_p_* is defined as follows:

(1) $$LDOF(xp)=\frac{{\bar{d}}_{xp}}{{\bar{D}}_{xp}}$$

Definition 1 (*KNN* distance of *x_p_*): Let *N_k_* be the set of the *k*-nearest neighbors of object *x_p_* (excluding *x_p_*). The *k*-nearest neighbors’ distance of *x_p_* equals the average distance from *x_p_* to all objects in *N_k_*. The *k*-nearest neighbors’ distance of object *x_p_* is defined as follows:

(2) $${\bar{d}}_{xp}=\frac{1}{k}\sum_{xi\in Nk}dist(x_{i},x_{p})$$

Definition 2 (*KNN* inner distance of *x_p_*): Given the *k*-nearest neighbors’ set *N_k_* of object *x_p_*, the *k*-nearest neighbors’ inner distance of *x_p_* is defined as the average distance among objects in *N_k_*:

(3) D¯xp=1k(k−1)∑xi,xi'∈Nk,i≠i'dist(xi,xi')

In our work, for a given set with *n* same-length SSE fragments, the *LDOF* value is a measure of how far outside its neighborhood system the fragment is. If the value ≥1, the fragments deviate from the neighborhood *k* fragments [33]; thus, any fragment with an *LDOF* value more than 1 was detected as an outlier. The detection precision of the method remains stable over a large range of *k* values, and the minimum value for *k* is 3× (length of the fragment); in our outlier fragment process, *k* is set to$\sqrt{n}$. The total number of outlier fragments for 21 SSEs is shown in Table 6.

### 3.4. Clustering and Central Poses Selection

To construct a central pose pool for (*s*, *l*, *n*) C_α_ fragments, *s* is the secondary structural feature assigned by DSSP, *l* is the length of the secondary structure and *n* is the total number of poses within the cluster. A five-step procedure was used to select the central poses. First, the secondary structural features for each residue in set **R** were automatically generated by DSSP. Second, each set of (*s*, *l*) C_α_ atom coordinates was extracted from PDB files. Thus, the fragment can be represented as an *l × 3* matrix, in which the *i*th row contains the coordinates of the *i*th C_α_ atom in the fragment. The *LDOF* factor was then used to detect outlier poses, which were excluded as unacceptable poses in the subsequent steps. Thereafter, our geometric clustering program was applied to cluster the C_α_ atom fragment sets with identical (*s*, *l*) coordinates [32]. Our algorithm is a top-down approach that recursively selects the outliers as seeds to form new clusters until all of the structures within a cluster satisfy a classification criterion (RMSD threshold). The criterion threshold for *l*-length C_α_ atom set clustering is *R*_max_, and our program was also applied to other clustering processes in the paper. Finally, the central pose in the cluster was selected as part of our central poses pool, and the central pose was defined as the pose with the minimum total RMSD with other poses within the cluster. The maximum RMSD between any pose in the cluster and the central pose was recorded, and the max RMSD value was used as the RMSD threshold for the subsequent SSE assignment. The RMSD between two paired sets of the same number of C_α_ atoms was calculated using the algorithm developed by Kabsch [37].

*R*_max_ determination: We first obtained the RMSD statistics for any two C_α_ fragments with the same (*s*, *l*). The statistical data were then fitted to the normal distribution. As shown in Table 6, the RMSD statistics for major SSEs fit a normal distribution very well, and the parameter μ was small except for left-handed helices and β-sheets with lengths of more than 5 residues. We used MATLAB to fit the data. The adjusted R-squared value accounts for the degrees of freedom, which indicates the goodness of fit (shown in Table 6). The adjusted *R*-squared statistic has a maximum value of 1, with a value closer to 1 indicating a better fit. In addition, the parameter μ was set to the *R*_max_ for the following subsequent step.

**Figure 4:**
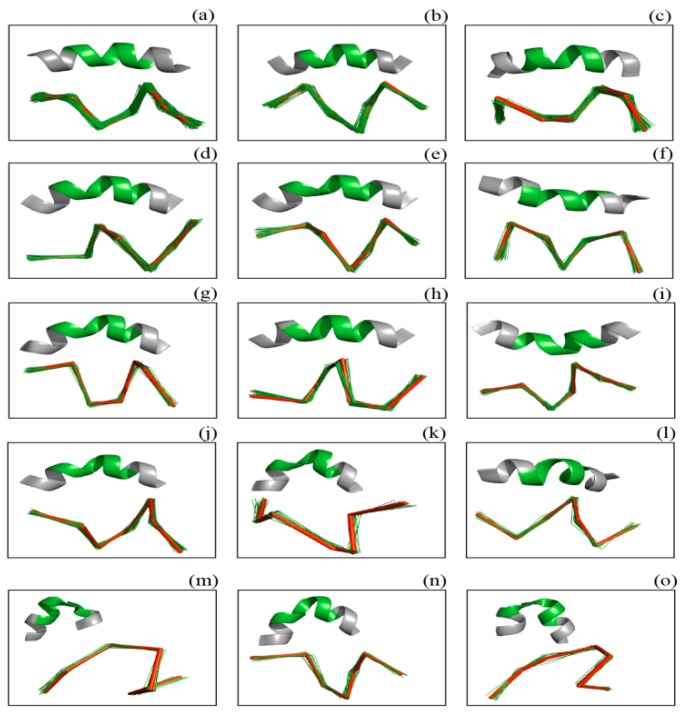
The clusters of α-helix bend fragments. (**a**–**o**) show 15 clusters after clustering α-helix bend fragments. The central fragments within clusters are displayed as red stick and green cartoon models, and the other fragments within clusters are displayed as green lines. We only show the odd clusters after the clusters were ordered by the number of fragments because this figure is an intuitive illustration of our algorithm.

Central α-helix bend fragment pool: A regular hydrogen bond pattern between the CO of residue *i* and the NH of residue *i*+4 results in a uniform α-helix in terms of rise of per residue, number of residues per turn and number of twists per turn. However, helix kinks and bends are common in long α-helices [38]. The longest α-helix in our pool was only 8 residues, and helices with kinks or bends are more likely to be classified as “rare poses” by DSSP. Because helix curves are visually allowed by crystallographers but tend to be detected as outlier and excluded, a merge step was developed to solve this problem. To merge two adjacent α-helices assigned by our program, we constructed a central α-helix bend pose pool: for residue *i* (residues from *i* − 5 to *i* + 5 should be categorized as α-helix by DSSP) with helix bending angles >20°, the seven consecutive C_α_ atoms from *i − 3* to *i + 3* were considered bend helix fragments. HELIX-F, a software program that can be applied to analyze protein helix geometry, was used to calculate the helix-bending angle for residues [39]. These 7-residue-length C_α_ atom sets were then clustered, whereas the RMSD threshold for clustering the helix bend poses was 0.5 Å. Subsequently, a total of 53 clusters were obtained, and the central poses in the top 20 clusters (ordered by number of fragments within the cluster) were selected as central α-helix bend fragments, the same as for other SSEs (Figure 4). The maximum RMSD between any fragments within the cluster and the central fragment was set to the threshold for new assignment in our algorithm.

Paired β-sheet ladder central pose pool: The ladders of paired residues were joined to form paired β-strands. In this pool, C_α_ ladder fragments in β-sheets are generated to pair two β-strand residues. The fragment consists of four C_α_ atoms linked by a pair of covalent bonds and a pair of hydrogen bonds. The DSSP output file was used to identify the paired β-sheet unit, *i.e.*, for two consecutive β-strand residues *i*, *i* + 1 with their parallel β-strand hydrogen bond partner *j*, *j* + 1, the C_α_ atoms of residue (*i*, *i* + 1, *j* + 1, *j*) were taken as a paired parallel β-sheet ladder fragment; residues *j* and *j* + 1 were also required to be assigned as β-sheet as by DSSP; their hydrogen bond partner information was obtained from the “BP1” and “BP2” columns of the DSSP output file. The clustering results for parallel and anti-parallel paired β-sheet ladder fragments are also shown in Table 6.

### 3.5. Our Secondary Structure Assignment Algorithm

#### 3.5.1. Helix Assignment

Let Set Pose (α-helix, length, *k*) be the pool of central poses for α-helices, and RMSD (α-helix, length, *k*) represents their corresponding largest distance threshold values; *k* is the cluster index.

First Step:


LET *a_i_* = 0, *i* = 0,…, n // All residues are initialized as coil
FOR *i* < *n*
FOR *len* (length from 8 to 4)
FOR *k* ∈ α-helix Central Pose Set Index
IF *dist* [Segment (*i*, *i* + *len*), Pose (α-helix, *len*, *k*)] < RMSD (α-helix, *len*, *k*) THEN
*a_(i +_ *_1, *i + len-*2)_ = 1 // Residues from *i* + 1 to *i* + len-2 are labeled as α-helix
END IF
END FOR
END FOR
END FOR


Second Step: The merge process of two adjacent α-helices:


FOR *i* < *n*
IF (*a_i_* == 0) AND (*a_i −_ *_1_ == 1) AND (*a_i +_* _3_ == 1) / Merge two adjacent helices less than four residues apart
FOR any seven consecutive residues including *i*, *i* + 1 and *i* + 2
FOR *k* ∈ Helix Kink Pose Set
IF *dist* [The seven residues fragment, Pose (helix kink, 7, *k*)] < RMSD (helix kink, 7, *k*)
*a_i_* = *a_i +_ *_1_ = *a_i +_* _2_ = 1 // Residues *i*, *i* + 1, *i* + 2 are label as α-helix
END IF
END FOR
END FOR
END IF
END FOR


The assignments of π-helices, 3_10_-helices, and left-handed helices are the same as for the first step of α-helices, with constant parameter lengths of 5, 3, 4, and 3, respectively. The priority of the three helix elements is π-helix > α-helix > 3_10_-helix; left-handed helices do not overlap with right-handed helices. We do not provide the merging process for these SSEs because their average lengths are 5.4, 3.3, 4.1, and 3.2, respectively, according to the DSSP assignment. In other words, 3_10_-helices, π-helices and left-handed helices with more than eight residues are rare.

#### 3.5.2. Parallel β-sheet Assignment

Let Set Pose (parallel β-strand, length, *k*) be the pool of parallel β-strand central poses, and RMSD (parallel β-strand, length, *k*) represents their corresponding largest distance threshold values between any other poses and the central pose in the same cluster; *k* is the cluster index.

First Step:


LET *b_i_* = 0, *i* = 0,…, *n* // All residues are initialized as coil
FOR *i* < *n*
FOR *len* (length from 5 to 4)
FOR *k* ∈ Parallel β-strand Pose Set Index
IF *dist* [Segment(*i*, *i + len*), Pose(β-strand, *len*, *k*)] < RMSD (Parallel β-strand, *len*, *k*) THEN
*b_(i, i + len-_*_1)_ =1 // Residues from *i* to *i* + len-1 are label as parallel β-strand
END IF
END FOR
END FOR
END FOR


Second Step: Matching the parallel β-sheet ladder between two β-strands.


FOR *i* < *n*
IF (*b_i_* == 1) AND (*b_i_ *_+ 1_ == 1) // Find residues have been assigned as β-strand
FOR *j* = 1 to *n* (*j* ≠ *i* − 1, *i*, *i +* 1) // Find the hydrogen bond partner β-strand residues
IF (*b_j_* > 0) AND (*b_j_ *_+ 1_ > 0)
FOR *k* ∈ Parallel β-sheet Ladder Pose Index
IF *dist* [Segment(*i*, *i* + 1, *j* + 1, *j*), Pose(ladder, 4 ,*k*)] < RMSD (ladder, 4, *k*) THEN
*b_i_*++,*b_i_ *_+ 1_++
END FOR
END IF
END FOR
END IF
END FOR


Finally, the β-strand residues that can form parallel β-sheet ladders with residues in other strands (*b_j_* > 1) are classified as parallel β-sheets. The difference between the assignment of antiparallel β-sheets and parallel β-sheet lies in the pose set selection: we selected an antiparallel β-strand pose set to identify parallel β-strand residues and an antiparallel β-sheet ladder pose set to identify partners of the antiparallel β-strand residues.

### 3.6. Comparison Measures

Overall agreement (*Q3* score): Different programs offer different classes of secondary structure; DSSP offers eight classes of secondary structures, whereas P-SEA only provides three secondary elements [6,12]. To evaluate the secondary structure agreement between different programs, we grouped all of the provided secondary features into three elements: helix, β-strand or coil. Detailed information on these conventions is shown in Table S3. The overall agreement *O*(*x*,*y*) is the percentage of residues assigned to the same element when comparing two different programs: *O*(*x*,*y*)* = N_id_/N_total_*, in which *N_id_* is the number of residues for which both programs *x* and *y* are identical, and *N_total_* is the total number of residues in a defined secondary structure [40].

The *SOV* score (Segment Overlap Score) described by Zemla was used to evaluate the agreement for segment *i* (helix, sheet, coil, *etc.*) assigned to two structures: the reference structure and the prediction structure [41]. The score depends on the structure that was selected as the reference and has been widely used to compare secondary structure assignment [13,17,42]. For element *i*, let (*S_1_*, *S_2_*) denote a pair of overlapping segments. The *SOV* is then defined as follows [40]:

(4) $$SOV(i)=100\times \frac{1}{N(i)}\sum_{S(i)}\left[\frac{minov(s_{1},s_{2})+\delta (s_{1},s_{2})}{maxov(s_{1},s_{2})}\times len(s_{1})\right]$$

in which *len*(*S*_1_) is the number of residues in segment *S*_1_, *minov*(*S*_1_,*S*_2_) is the length of the actual length of the overlap between *S*_1_ and *S*_2_ in element *i*, and *maxov*(*S*_1_,*S*_2_) is the total extent of either *S*_1_ or *S*_2_ to have a residue in element *i*. The normalization value, *N*(*i*), is defined as follows:

(5) $$N(i)=\sum_{S(i)}len(s_{1})+\sum_{S'(i)}len(s_{1})$$

The first sum in the above expression is taken over all the segment pairs in state *i* that overlap by at least one residue; the second sum is taken over the remaining segments in state *i* found in the reference assignment.

$\delta (s_{1},s_{2})$ is defined as follows:

(6) $$\delta (s_{1},s_{2})=min\left\{\begin{array}{l} maxov(S_{1},S_{2})-minov(S_{1},S_{2})\\ minov(S_{1},S_{2})\\ int(\frac{len(S_{1})}{2})\\ int(\frac{len(S_{2})}{2}) \end{array}\right\}$$

### 3.7. Secondary Structure Assignment Methods in Comparison

In total, we obtained 10 SSE assignment methods that are available on the Internet or by asking the authors directly (Table S4).

## 4. The Correlation between Outlier Poses Assigned by DSSP and Protein–Ligand Binding Sites

**Figure 5:**
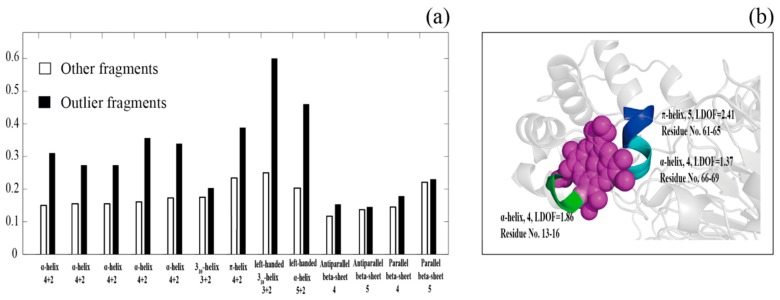
A histogram of the correlation between protein–ligand binding sites and two types of fragments: outlier fragments (black bar) and other fragments (white bar). (**a**) shows a histogram of the two types of fragments *vs.* a protein-ligand binding site. The *x*-axis is their secondary structure feature and length, while the *y*-axis is the probability of the secondary structure observed at the protein-ligand binding site (distance less than 4Å). Figure b shows an example illustrating the outlier poses detected at protein–ligand binding sites: for cytochrome cd1 nitrite reductase (pdb ID: 1qks), there are three outlier helix fragments (colored green, blue and cyan) around the binding site (the ligand is colored in purple). The *LDOF* values and residue index for the helix fragments are also labeled in figure **b**.

The first step of our method consists of detecting outlier poses from secondary structure segments with the same length assigned by DSSP. As described above, poses with high *LDOF* values were selected as outliers, which are used to elucidate structure–function relationships by identifying structure–function differences between the outlier poses and other poses. Among the 9898 structures in set **A**, in total, 4716 proteins contain at least one ligand. Using the 4716 structures, we classified the SSEs into two classes: outlier poses, poses within clusters. We then computed their probability of being observed at the protein–ligand binding site (distance less than 4 Å). The distance between a ligand and an SSE fragment was defined as the shortest distance between any ligand atom and any atom that belongs to the SSE fragment residues. Notably, metal ions and inorganic anions, such as Na^+^, Ca^2+^, Cl^−^, PO_4_^3−^ and SO_4_^2−^, were excluded from our definition of ligands. As shown in Figure 5, outlier α-helix and π-helix poses are more likely to be observed at protein–ligand binding sites; the probability of a left-handed 3_10_-helix and a left-handed α-helix being detected at a protein–ligand binding site is also higher than that of other poses. However, outlier poses in 3_10_-helices and β-sheets do not show preference at protein–ligand binding sites. Furthermore, as shown in Figure 5b, three outlier poses were in the protein–ligand binding site (porphyrin binding site). The result shows that outlier poses, especially outlier helices, perform different structural functions than remaining fragments. This correlation should be useful for discovering structure–function relationships in proteins.

## 5. Conclusions

Making uniform secondary structure assignments is an important task. Dozens of programs have been developed since DSSP was released in 1983, but DSSP remains the “gold standard” of secondary structure assignment. Compared with another popular program, STRIDE, our method aims to make C_α_ fragments more uniform instead of only using local ϕ/ψ torsion angle criteria. Moreover, three subtle helices were also detected using our algorithm: 3_10_-helices, π-helices and left-handed 3_10_-helices. Hydrogen bond energy calculations are limited because the calculation is empirical and features many overlaps for *i* + 3, *i* + 4, and *i* + 5 hydrogen bond patterns. Our method can be considered a knowledge-based secondary structure assignment program from C_α_ fragments assigned by DSSP. Rare fragments can be detected using our outlier fragments detection. In a large-scale comparison of 11 available methods, PCASSO agrees most with DSSP, followed by SACF, KAKSI and PROSS, with both PCASSO and SACF preferring to reduce residues at the N cap and C cap regions of helices and β-sheets if DSSP is taken as the standard method. The helix outlier fragments detected by our method perform very different biological functions in the identified proteins. The structurally uniform SSEs assigned by our method should be useful for protein classification and prediction.

## Figures and Tables

**Table 1:** The agreement (%) of eleven programs on set **T**. The agreement percentage was computed using *Q3* score.

Method	Dssp	Stride	P-sea	Kaksi	Disicl	Palsse	Segno	Pross	Xtlsstr	Pcasso
Sacf	84.7	85.1	81.8	82.6	76.9	68.4	80.5	83.1	76.1	84.3
Dssp		95.0	80.9	83.5	78.9	72.9	83.0	84.3	77.2	93.5
Stride			81.1	84.1	78.4	73.6	82.5	84.8	80.2	92.0
P-sea				82.3	78.3	68.8	85.9	86.2	74.4	82.1
Kaksi					74.8	77.5	80.5	82.9	78.5	83.8
Disicl						63.1	80.8	81.8	74.9	79.6
Palsse							66.3	66.1	70.6	73.6
Segno								87.4	76.4	82.4
Pross									79.3	84.5
Xtlsstr										79.2

**Table 2:** SOV scores (%) between any two of the eleven programs on Set **T** for helix. For every SOV score in the table, the corresponding method in the first column is taken as the reference method.

Method	Sacf	Dssp	Stride	P-sea	Kaksi	Disicl	Palsse	Segno	Pross	Xtlsstr	Pcasso
Sacf		96.6	94.1	92.6	92.6	88.3	81.7	80.1	91.2	90.3	95.2
Dssp	91.3		93.7	86.0	88.4	82.9	81.1	75.8	86.1	89.2	94.1
Stride	90.1	95.2		86.2	88.0	84.4	82.5	77.1	87.4	92.6	92.7
P-sea	96.9	96.7	94.2		95.7	91.3	84.1	83.7	95.2	91.6	96.5
Kaksi	93.8	96.0	93.4	92.6		84.7	86.3	79.1	92.8	91.6	95.0
Disicl	87.3	89.9	89.6	85.6	85.7		72.8	80.0	87.6	85.6	89.6
Palsse	60.3	62.4	63.1	63.7	67.1	47.8		50.5	62.2	69.0	59.7
Segno	92.9	94.1	93.2	92.3	91.5	94.4	76.8		93.5	89.5	94.1
Pross	95.9	97.4	97.5	95.6	96.7	93.9	83.1	86.2		93.9	97.1
Xtlsstr	82.7	86.8	89.1	81.5	83.9	76.7	85.6	71.7	81.9		84.5
Pcasso	90.9	96.4	93.2	87.6	89.5	84.7	80.3	77.4	87.6	89.4	

**Table 3:** *SOV* scores (%) between any two of eleven methods on Set **T** for β-sheet. For every *SOV* score in the table, the corresponding method in the first column is taken as the reference method.

Method	Sacf	Dssp	Stride	P-sea	Kaksi	Disicl	Palsse	Segno	Pross	Xtlsstr	Pcasso
Sacf		86.0	85.4	78.7	86.0	78.3	68.9	80.9	78.6	71.3	87.1
Dssp	81.2		97.0	78.0	88.0	70.8	73.1	80.4	77.2	71.3	89.2
Stride	79.4	96.7			87.3	70.2	73.3	80.2	75.7	70.7	87.9
P-sea	78.7	78.9	78.6		83.2	77.2	70.8	87.0	79.0	68.7	80.6
Kaksi	84.5	92.0	91.6	83.8		77.0	76.9	86.4	80.6	73.0	91.9
Disicl	64.6	68.6	68.7	71.0	69.6		53.4	75.7	72.8	65.3	70.8
Palsse	45.7	51.3	51.6	50.4	52.1	35.8		48.9	43.4	43.0	47.5
Segno	75.8	79.8	79.9	82.5	82.3	80.9	68.3		81.5	72.9	81.2
Pross	81.7	83.1	83.4	83.2	84.4	88.9	64.8	91.2		76.1	84.5
Xtlsstr	74.9	77.8	77.9	73.8	77.2	79.1	62.9	82.8	78.0		77.0
Pcasso	84.2	90.8	89.7	78.8	87.5	73.0	70.3	81.1	76.5	70.3	

**Table 4:** Discrepancies between terminals in the helices assigned by DSSP and other methods.

Method	Same	N cap		N cap		C cap		C cap	
		+(1–2)	+(>2)	−(1–2)	−(>2)	+(1-2)	+(>2)	−(1–2)	−(>2)
Sacf	5194	1407	23	1919	534	1865	15	3142	578
Stride	11,388	990	34	332	80	801	60	401	62
P-sea	1639	4782	678	870	569	4405	610	1267	423
Kaksi	1761	5765	153	2269	217	5347	131	1737	92
Disicl	1310	4090	252	1828	369	1131	96	7306	587
Palsse	87	7423	726	121	59	7153	728	121	26
Segno	2734	5222	448	913	332	3344	397	1182	253
Pross	3037	2626	117	1638	796	2350	107	2326	592
Xtlsstr	803	5932	332	1855	600	1173	130	4023	857
Pcasso	5950	1211	50	1856	347	1795	35	2302	272

The second column shows the number of helices assigned by a given method (first column) that are identical to the helices assigned by DSSP. The third through tenth columns show the helices assigned by DSSP with at most one or two residues difference (1–2 residues) or more than two residue (>2 residues) divergence with the method in the first column. Note that a helix assigned by other methods can disagree with DSSP at both the N cap and C cap. “+”, a helix assigned by another method has more residues at the N or C cap than the helix assigned by DSSP; “−”, a helix assigned by another method has fewer residues at the N or C cap region than the helix assigned by DSSP.

**Table 5:** Discrepancies between N and C caps in the β-sheets assigned by DSSP and other methods.

Method	Same	N cap		N cap		C cap		C cap	
		+(1–2)	+(>2)	−(1–2)	−(>2)	+(1–2)	+(>2)	−(1–2)	−(>2)
Sacf	2375	1355	16	2218	535	1902	11	2,897	578
Stride	8352	733	83	285	80	544	69	353	63
P-sea	1621	3260	568	853	486	3267	473	1,225	433
Kaksi	1473	4138	71	2163	317	3890	73	1,638	195
Disicl	815	2720	182	1602	371	749	85	5,367	591
Palsse	56	5713	786	116	63	5513	781	114	28
Segno	2364	3753	384	851	337	2322	335	1085	255
Pross	2481	1820	83	1567	802	1544	84	2200	594
Xtlsstr	636	4447	275	1791	602	829	124	3507	863
Pcasso	4994	867	66	1267	348	973	48	1490	273

The second column shows the number of strands in β-sheets assigned by a given method (first column) that are identical to the strands assigned by DSSP. The third through tenth columns show the strands in β-sheets assigned by DSSP with at most one or two residues different (1–2 residues) or a more than two residue (>2 residues) divergence with the method in the first column. Note that strands in β-sheets assigned by other methods can disagree with DSSP at both the N cap and C cap. “+”, a strand assigned by another method has more residues at the N or C cap than the strand assigned by DSSP; “−”, a strand assigned by another method has fewer residues at the N or C cap than the strand assigned by DSSP.

**Table 6:** The normal distribution parameters and clustering information for 21 secondary structure elements.

SSE Name	Len	μ (Å) ^1^	Σ ^2^	Adj.R-Square	Total Number of SSEs	Number of Outliers	Number of Clusters	Max ^3^
α-helix	4 + 2^4^	0.411	0.218	0.969	4776	496	18	682
α-helix	5 + 2	0.388	0.173	0.971	2842	349	25	276
α-helix	6 + 2	0.393	0.150	0.979	3159	357	28	315
α-helix	7 + 2	0.418	0.185	0.976	3578	326	33	383
α-helix	8 + 2	0.435	0.189	0.970	3521	563	25	273
3_10_-helix	3 + 2	0.303	0.157	0.980	15,689	2334	32	1830
π-helix	5 + 2	0.516	0.437	0.955	1243	224	19	304
Left-α-helix	4 + 2	1.012	8.004	0.815	72	23	8	16
Left-3_10_-helix	3 + 2	0.596	0.239	0.898	812	211	21	82
Parallel β-ladder	4	0.352	0.314	0.987	62,204	6917	22	7821
Antiparallel β-ladder	4	0.427	0.201	0.989	97,088	8562	23	8787
Parallel β-strand	4	0.383	0.189	0.999	5374	689	25	878
Parallel β-strand	5	0.496	0.486	0.973	5846	858	28	664
Parallel β-strand	6	0.776	0.579	0.966	4678	868	31	670
Parallel β-strand	7	1.400	0.623	0.898	2608	419	28	385
Parallel β-strand	8	1.631	1.282	0.921	1627	37	32	337
Antiparallel β-strand	4	0.543	0.571	0.984	6176	821	19	1048
Antiparallel β-strand	5	0.546	0.671	0.959	6554	867	28	886
Antiparallel β-strand	6	1.367	1.825	0.926	4600	672	25	738
Antiparallel β-strand	7	1.882	0.817	0.943	5217	841	26	909
Antiparallel β-strand	8	1.994	0.824	0.945	4221	898	31	682

^1^ Expectation value of the *dist* distribution*.* The statistics of *dist* is fitted to a normal distribution while *dist* is the RMSD between any two of the fragments of same length (column 2) and secondary structure (column 1); ^2^ Variance of the *dist* distribution; ^3^ Number of fragments in the largest cluster; ^4^ For a DSSP assigned helix composed of *n* residues, we extend one residue at both N and C terminal of the helix since the two residues also form hydrogen bond with the residues in the helix, thus the finally length of the helix is *n* + 2.

## Supplementary Materials

Supplementary materials can be found at http://www.mdpi.com/1422-0067/17/3/333/s1.

## References

[1] Pauling L., Corey R.B., Branson H.R. (1951). The structure of proteins; two hydrogen-bonded helical configurations of the polypeptide chain. Proc. Natl. Acad. Sci. USA.

[2] Vieira-Pires R.S., Morais-Cabral J.H. (2010). 3(10) helices in channels and other membrane proteins. J. Gen. Physiol..

[3] Wilmot C.M., Thornton J.M. (1990). β-turns and their distortions: A proposed new nomenclature. Protein Eng..

[4] Richardson J.S., Getzoff E.D., Richardson D.C. (1978). The β bulge: A common small unit of nonrepetitive protein structure. Proc. Natl Acad. Sci. USA.

[5] Hutchinson E.G., Thornton J.M. (1996). Promotif—A program to identify and analyze structural motifs in proteins. Protein Sci..

[6] Labesse G., Colloc’h N., Pothier J., Mornon J.P. (1997). P-sea: A new efficient assignment of secondary structure from c alpha trace of proteins. Comput. Appl. Biosci..

[7] Richardson J.S. (1985). Schematic drawings of protein structures. Methods Enzymol..

[8] Sillitoe I., Lewis T.E., Cuff A., Das S., Ashford P., Dawson N.L., Furnham N., Laskowski R.A., Lee D., Lees J.G. (2015). Cath: Comprehensive structural and functional annotations for genome sequences. Nucleic Acids Res..

[9] Sali A., Blundell T.L. (1990). Definition of general topological equivalence in protein structures. A procedure involving comparison of properties and relationships through simulated annealing and dynamic programming. J. Mol. Biol..

[10] Hubbard T., Tramontano A. (1996). Update on protein structure prediction: Results of the 1995 irbm workshop. Fold. Des..

[11] Levitt M., Greer J. (1977). Automatic identification of secondary structure in globular proteins. J. Mol. Biol..

[12] Kabsch W., Sander C. (1983). Dictionary of protein secondary structure: Pattern recognition of hydrogen-bonded and geometrical features. Biopolymers.

[13] Konagurthu A.S., Lesk A.M., Allison L. (2012). Minimum message length inference of secondary structure from protein coordinate data. Bioinformatics.

[14] Berman H.M., Westbrook J., Feng Z., Gilliland G., Bhat T.N., Weissig H., Shindyalov I.N., Bourne P.E. (2000). The protein data bank. Nucleic Acids Res..

[15] Frishman D., Argos P. (1995). Knowledge-based protein secondary structure assignment. Proteins.

[16] Fodje M.N., Al-Karadaghi S. (2002). Occurrence, conformational features and amino acid propensities for the pi-helix. Protein Eng..

[17] Martin J., Letellier G., Marin A., Taly J.F., de Brevern A.G., Gibrat J.F. (2005). Protein secondary structure assignment revisited: A detailed analysis of different assignment methods. BMC Struct. Biol..

[18] King S.M., Johnson W.C. (1999). Assigning secondary structure from protein coordinate data. Proteins.

[19] Majumdar I., Krishna S.S., Grishin N.V. (2005). Palsse: A program to delineate linear secondary structural elements from protein structures. BMC Bioinform..

[20] Park S.Y., Yoo M.J., Shin J., Cho K.H. (2011). Saba (secondary structure assignment program based on only alpha carbons): A novel pseudo center geometrical criterion for accurate assignment of protein secondary structures. BMB Rep..

[21] Srinivasan R., Rose G.D. (1999). A physical basis for protein secondary structure. Proc. Natl Acad. Sci. USA.

[22] Cubellis M.V., Cailliez F., Lovell S.C. (2005). Secondary structure assignment that accurately reflects physical and evolutionary characteristics. BMC Bioinform..

[23] Nagy G., Oostenbrink C. (2014). Dihedral-based segment identification and classification of biopolymers i: Proteins. J. Chem. Inform. Model..

[24] Law S.M., Frank A.T., Brooks C.L. (2014). Pcasso: A fast and efficient c alpha-based method for accurately assigning protein secondary structure elements. J. Comput. Chem..

[25] Richards F.M., Kundrot C.E. (1988). Identification of structural motifs from protein coordinate data: Secondary structure and first-level supersecondary structure. Proteins.

[26] Taylor W.R. (2001). Defining linear segments in protein structure. J. Mol. Biol..

[27] Sklenar H., Etchebest C., Lavery R. (1989). Describing protein structure: A general algorithm yielding complete helicoidal parameters and a unique overall axis. Proteins.

[28] Zhang W., Dunker A.K., Zhou Y.Q. (2008). Assessing secondary structure assignment of protein structures by using pairwise sequence-alignment benchmarks. Proteins.

[29] Zacharias J., Knapp E.W. (2014). Protein secondary structure classification revisited: Processing dssp information with pssc. J. Chem. Inform. Model..

[30] Hodge V.J., Austin J. (2004). A survey of outlier detection methodologies. Artif. Intell. Rev..

[31] Jain A.K. (2010). Data clustering: 50 years beyond k-means. Pattern Recognit. Lett..

[32] Xu S., Zou S., Wang L. (2015). A geometric clustering algorithm with applications to structural data. J. Comput. Biol..

[33] Zhang K., Hutter M., Jin H.D. (2009). A new local distance-based outlier detection approach for scattered real-world data. Data Min. Knowl. Discov..

[34] Colloc'h N., Etchebest C., Thoreau E., Henrissat B., Mornon J.P. (1993). Comparison of three algorithms for the assignment of secondary structure in proteins: The advantages of a consensus assignment. Protein Eng..

[35] Berman H., Henrick K., Nakamura H., Markley J.L. (2007). The worldwide protein data bank: Ensuring a single, uniform archive of PDB data. Nucleic Acids Res..

[36] Novotny M., Kleywegt G.J. (2005). A survey of left-handed helices in protein structures. J. Mol. Biol..

[37] Kabsch W. (1978). A discussion of the solution for the best rotation to relate two sets of vectors. Acta Crystallogr. Sect. A.

[38] Wilman H.R., Shi J., Deane C.M. (2014). Helix kinks are equally prevalent in soluble and membrane proteins. Proteins.

[39] Cao C., Xu S., Wang L. (2015). An algorithm for protein helix assignment using helix geometry. PLoS ONE.

[40] Zemla A., Venclovas C., Fidelis K., Rost B. (1999). A modified definition of sov, a segment-based measure for protein secondary structure prediction assessment. Proteins.

[41] Rost B., Sander C., Schneider R. (1994). Redefining the goals of protein secondary structure prediction. J. Mol. Biol..

[42] Matsuo K., Watanabe H., Gekko K. (2008). Improved sequence-based prediction of protein secondary structures by combining vacuum-ultraviolet circular dichroism spectroscopy with neural network. Proteins.

