# Supplementary material for: A New Secondary Structure Assignment Algorithm Using Cα Backbone Fragments
Source: Int J Mol Sci. 2016 Mar 11;17(3):333. doi: 10.3390/ijms17030333 (PMC4813195; doi:10.3390/ijms17030333)
Supplement: Supplementary file 1 [file ijms-17-00333-s001.pdf]

Supplementary Materials: A New Secondary Structure Assignment Algorithm Using Cα Backbone Fragments

Chen Cao, Guishen Wang, An Liu, Shutan Xu, Lincong Wang and Shuxue Zou

Table S1. The agreement of 11 secondary structure assignment methods on set L. The agreement percentage was computed using the Q3 score.

Method	Dssp	Stride	P-sea	Kaksi	Disicl	Palsse	Segno	Pross	Xtlsstr	Pcasso
Sasf	84.5	84.3	81.8	84.0	76.2	67.3	80.2	83.5	75.5	84.4
Dssp		93.6	81.4	84.5	78.1	73.1	81.6	84.5	78.2	88.1
Stride			82.7	85.6	78.0	75.2	81.1	85.0	78.3	89.6
P-sea				82.4	76.5	71.9	82.4	83.2	72.8	83.8
Kaksi					75.8	75.7	80.4	84.0	77.8	84.8
Disicl						61.7	81.2	81.5	74.2	77.8
Palsse							66.1	65.6	65.1	76.5
Segno								85.7	75.3	81.1
Pross									79.7	83.5
Xtlsstr										76.7

Table S2. The agreement of nine secondary structure assignment methods on set N. The agreement percentage was computed using the Q3 score. DISICL and SEGNO could not assign most proteins in set N for unknown reasons.

Method	Dssp	Stride	P-sea	Kaksi	Palsse	Pross	Xtlsstr	Pcasso
Sasf	84.3	85.6	78.3	83.2	69.5	81.9	76.6	85.1
Dssp		94.3	80.1	83.8	71.5	84.3	76.8	90.6
Stride			81.4	85.2	74.1	84.8	77.1	90.7
P-sea				80.3	67.8	85.3	75.3	80.7
Kaksi					73.4	83.6	77.9	84.2
Palsse						65.8	67.7	72.7
Pross							78.0	83.7
Xtlsstr								75.0

Table S3. Converting SSEs assigned by different programs into three states: H, E, C. "LH" is the abbreviation for a left-handed helix, whereas "AE" and "PE" are the abbreviations of antiparallel β-sheets and parallel β-sheets, respectively.

Method	H	E	C
SACF	H, G, I, LH	AE, PE	C, B, P
DSSP	H, G, I	E	B, T, S, C
STRIDE	H, G, I	E	B, T, C
P-SEA	a	b	c
PALSSE	HELIX	SHEET	" "
KAKSI	H	b	"."
DISICL	3HT, HEL	BS	IRB, BT, OTT, LHT, UC
PROSS	H	E	T, P
SEGNO	H,G,I	E, e	B, b, P, p, O
XTLSSTR	H, h, G, g	E, e	T, P, "-", U
PCASSO	H	E	C

Table S4. Sources of 11 secondary structure assignment programs. We could not obtain other methods such as DEFINE, STICK, and SABA, as these programs are not available on the Internet.

DSSP	Available from http://swift.cmbi.ru.nl/gv/dssp/
STRIDE	Available from http://webclu.bio.wzw.tum.de/stride/
KAKSI	Available from http://www.dsimb.inserm.fr/~debrevern/TOOLS/KAKSI/
PBs	Available from Prof. Alexandre G de Brevern directly
P-SEA	Available from http://biopython.org/DIST/docs/api/Bio.PDB.PSEA-module.html
PCASSO	Available from http://brooks.chem.lsa.umich.edu/software
SEGNO	Available from Prof. Simon Lovell directly
PALSSE	Available from http://prodata.swmed.edu/palsse/palsse.php
XTLSSTR	Available from http://oregonstate.edu/dept/biochem/faculty/johnson.html
PROSS	Available from http://folding.chemistry.msstate.edu/utils/pross.html
DISICL	Available from http://disicl.boku.ac.at/

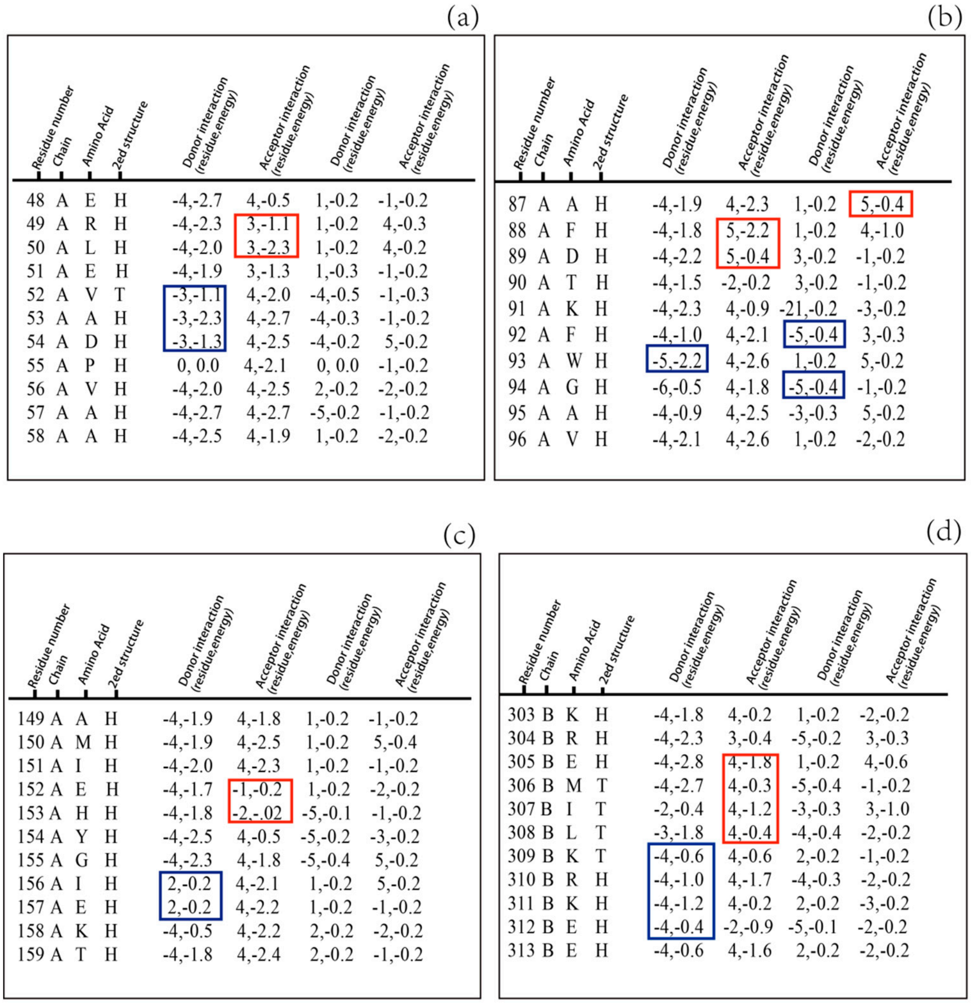

(a)

Residue number	Chain	Amino Acid	2ed structure	Donor interaction (residue,energy)	Acceptor interaction (residue,energy)	Donor interaction (residue,energy)	Acceptor interaction (residue,energy)
48	A	E	H	-4,-2.7	4,-0.5	1,-0.2	-1,-0.2
49	A	R	H	-4,-2.3	3,-1.1	1,-0.2	4,-0.3
50	A	L	H	-4,-2.0	3,-2.3	1,-0.2	4,-0.2
51	A	E	H	-4,-1.9	3,-1.3	1,-0.3	-1,-0.2
52	A	V	T	-3,-1.1	4,-2.0	-4,-0.5	-1,-0.3
53	A	A	H	-3,-2.3	4,-2.7	-4,-0.3	-1,-0.2
54	A	D	H	-3,-1.3	4,-2.5	-4,-0.2	5,-0.2
55	A	P	H	0, 0.0	4,-2.1	0, 0.0	-1,-0.2
56	A	V	H	-4,-2.0	4,-2.5	2,-0.2	-2,-0.2
57	A	A	H	-4,-2.7	4,-2.7	-5,-0.2	-1,-0.2
58	A	A	H	-4,-2.5	4,-1.9	1,-0.2	-2,-0.2

(b)

Residue number	Chain	Amino Acid	2ed structure	Donor interaction (residue,energy)	Acceptor interaction (residue,energy)	Donor interaction (residue,energy)	Acceptor interaction (residue,energy)
87	A	A	H	-4,-1.9	4,-2.3	1,-0.2	5,-0.4
88	A	F	H	-4,-1.8	5,-2.2	1,-0.2	4,-1.0
89	A	D	H	-4,-2.2	5,-0.4	3,-0.2	-1,-0.2
90	A	T	H	-4,-1.5	-2,-0.2	3,-0.2	-1,-0.2
91	A	K	H	-4,-2.3	4,-0.9	-21,-0.2	-3,-0.2
92	A	F	H	-4,-1.0	4,-2.1	-5,-0.4	3,-0.3
93	A	W	H	-5,-2.2	4,-2.6	1,-0.2	5,-0.2
94	A	G	H	-6,-0.5	4,-1.8	-5,-0.4	-1,-0.2
95	A	A	H	-4,-0.9	4,-2.5	-3,-0.3	5,-0.2
96	A	V	H	-4,-2.1	4,-2.6	1,-0.2	-2,-0.2

(c)

Residue number	Chain	Amino Acid	2ed structure	Donor interaction (residue,energy)	Acceptor interaction (residue,energy)	Donor interaction (residue,energy)	Acceptor interaction (residue,energy)
149	A	A	H	-4,-1.9	4,-1.8	1,-0.2	-1,-0.2
150	A	M	H	-4,-1.9	4,-2.5	1,-0.2	5,-0.4
151	A	I	H	-4,-2.0	4,-2.3	1,-0.2	-1,-0.2
152	A	E	H	-4,-1.7	-1,-0.2	1,-0.2	-2,-0.2
153	A	H	H	-4,-1.8	-2,-.02	-5,-0.1	-1,-0.2
154	A	Y	H	-4,-2.5	4,-0.5	-5,-0.2	-3,-0.2
155	A	G	H	-4,-2.3	4,-1.8	-5,-0.4	5,-0.2
156	A	I	H	2,-0.2	4,-2.1	1,-0.2	5,-0.2
157	A	E	H	2,-0.2	4,-2.2	1,-0.2	-1,-0.2
158	A	K	H	-4,-0.5	4,-2.2	2,-0.2	-2,-0.2
159	A	T	H	-4,-1.8	4,-2.4	2,-0.2	-1,-0.2

(d)

Residue number	Chain	Amino Acid	2ed structure	Donor interaction (residue,energy)	Acceptor interaction (residue,energy)	Donor interaction (residue,energy)	Acceptor interaction (residue,energy)
303	B	K	H	-4,-1.8	4,-0.2	1,-0.2	-2,-0.2
304	B	R	H	-4,-2.3	3,-0.4	-5,-0.2	3,-0.3
305	B	E	H	-4,-2.8	4,-1.8	1,-0.2	4,-0.6
306	B	M	T	-4,-2.7	4,-0.3	-5,-0.4	-1,-0.2
307	B	I	T	-2,-0.4	4,-1.2	-3,-0.3	3,-1.0
308	B	L	T	-3,-1.8	4,-0.4	-4,-0.4	-2,-0.2
309	B	K	T	-4,-0.6	4,-0.6	2,-0.2	-1,-0.2
310	B	R	H	-4,-1.0	4,-1.7	-4,-0.3	-2,-0.2
311	B	K	H	-4,-1.2	4,-0.2	2,-0.2	-3,-0.2
312	B	E	H	-4,-0.4	-2,-0.9	-5,-0.1	-2,-0.2
313	B	E	H	-4,-0.6	4,-1.6	2,-0.2	-2,-0.2

Figure S1. The hydrogen bond energy used by DSSP for secondary structure assignment. The data were obtained by DSSP for the proteins 2fp1 (**a**), 3ucf (**b**), 1q35 (**c**) and 1kb4 (**d**). The red rectangles indicate donor interactions, and the blue rectangles denote acceptor interactions. A careful examination of the hydrogen bond energies (kcal/mol) for these residues, as indicated by the rectangles, suggests that they could also be assigned to a 310-helix (residues 50–52, 2fp1) and π-helix (residues 89–93, 3ucf), even by the DSSP standard. As indicated in Figure 4c, an obvious (*i*,*i* + 4) hydrogen bond break forms the kink. The overlaps in hydrogen bond pattern hinder the assignment of secondary structures using only empirically based hydrogen bond energy, and our method can make the assignment more visually acceptable.
